# Software-Based Simulation on a 3D Environment for Vaccination Teaching and Learning: Design Science Research

**DOI:** 10.2196/35712

**Published:** 2022-12-02

**Authors:** Daniel Domingueti, Darlinton Barbosa Feres Carvalho, Diego Roberto Colombo Dias, Valéria Conceição Oliveira

**Affiliations:** 1 Departamento de Ciência da Computação Universidade Federal de São João del-Rei Sao Joao del Rei Brazil; 2 Campus Centro Oeste Dona Lindu Universidade Federal de São João del-Rei Divinopolis Brazil

**Keywords:** software simulation, vaccination room, immunization, teaching, training, evaluation, virtual world, Unity3D, SUS, UTAUT2

## Abstract

**Background:**

Student training requires specific laboratories for vaccination practice, which are usually limited, and even professionals’ continuing education regularly lacks proper care. Thus, new methodologies, concepts, and technologies, such as software-based simulations, are in highly demand.

**Objective:**

This work aims to develop a 3D virtual environment to support teaching activities in the vaccination room. The software-based simulation must contribute positively to teaching considering a variable set of scenarios.

**Methods:**

We applied the design science research method to guide the work. First, the concepts and opportunities were raised, which we used to build the simulation (ie, the proposed technological artifact). The development was assisted by a specialist, in which we sought to create a vaccination room according to Brazilian standards. The artifact evaluation was achieved in 2 stages: (1) an evaluation to validate the design with experts through the Delphi method; and (2) a field evaluation with nursing students to validate aspects of usability (System Usability Scale [SUS]) and technology acceptance and use (Unified Theory of Acceptance and Use of Technology version 2).

**Results:**

We built the simulation software using the Unity game engine. An additional module was also developed to create simulation scenarios and view the students’ performance reports. The design evaluation showed that the proposed solution is adequate. Students’ evaluations confirm good usability (SUS score of 81.4), besides highlighting Performance Expectation as the most positively influential factor of Behavioral Intention. Effort Expectancy is positively affected by younger users. Both evaluation audiences cited the high relevance of the proposed artifact for teaching. Points for improvement are also reported.

**Conclusions:**

The research accomplished its goal of creating a software-based simulation to support teaching scenarios in the vaccination room. The evaluations still reveal desirable improvements and user behavior toward this kind of technological artifact.

## Introduction

There are still few technologies to support teaching in the vaccination room. As it needs a specific laboratory, the education is typically related to managing the room and vaccine administration. Unfortunately, this is a common situation in Brazil. Despite having laboratories in (Brazilian) universities, learning usually happens in a professional environment with a trained nurse. Besides, in classrooms, the students may not be aware of the variety of situations that they will be encountering when interacting with a patient in the vaccination room.

In Brazil, as defined by the National Program of Immunizations (PNI), the team responsible for performing all the activities in the vaccination room comprises a nurse and nursing technicians or auxiliary nurses. PNI also lists the nurse’s tasks, such as preparing the vaccination room, performing the vaccination process, writing reports, providing the team’s continuing education process, and others. To sum up, the nurse is responsible for the overall supervision of the vaccination room [1].

Given the complexity observed in the tasks and the higher turnover rates of professionals, we need to highlight the importance of the team’s continuing education process. Although highly relevant, it is happening in scarcity and irregular ways. Nursing technicians or auxiliary nurses usually only receive training when they start their activities and when offered to senior professionals, mainly to update the vaccination schedule [2].

In this context, the proposal of new education and training methodologies based on integrating and participatory teaching and learning models is in high demand [3]. For instance, digital games and simulations allow the creation of virtual environments where players can learn about educational content. Games can generally motivate and engage students in learning activities [4]. More specifically, a simulation can benefit from the modern digital games tools and concepts to create more immersive educational experiences.

Although they share similar tools and ideas, digital games and simulations differ slightly. Games usually focus on the fun and competition among players, whereas simulations might focus on other objectives, such as those shared with an educational process. In a simulation, the essential elements from a process or event are replicated in a digital environment to promote an educational experience [5,6]. In this case, all elements essential to being represented in the simulation are considered: an application built to mirror—more accurately as possible—the object’s life cycle, process, or event [7].

Hoping to contribute to this sense, we evaluated the software development process for simulation to assist nursing students’ educational process in Brazil. Our hypothesis was that a 3D virtual environment, following standards and allowing the simulation of relevant scenarios, will be a viable alternative and positively contribute to teaching in the vaccination room. Therefore, our objective was to create a software-based simulation to assist nursing students’ educational process in Brazil. The software uses the concepts and tools of simulations and digital games applied to the vaccination room.

## Methods

### Study Design

We developed this research using the design science research (DSR) method. DSR is a method in which a designer answers relevant research questions through artifact creation. Essentially a problem-solving paradigm, DSR purposes innovative artifacts in which information systems become more efficient at solving relevant problems through a rigorous scientific process [8].

DSR starts by setting the basic requirements, problems, and opportunities. Later, the process follows the project research, in which the artifacts are designed, created, and their design further evaluated. The artifacts should be based on well-defined support theories or professional expertise. The process goes on with evaluations to assess the solution created. The researcher must communicate their results contributing to the scientific knowledge base at the end of the process [8,9].

**Figure 1:**
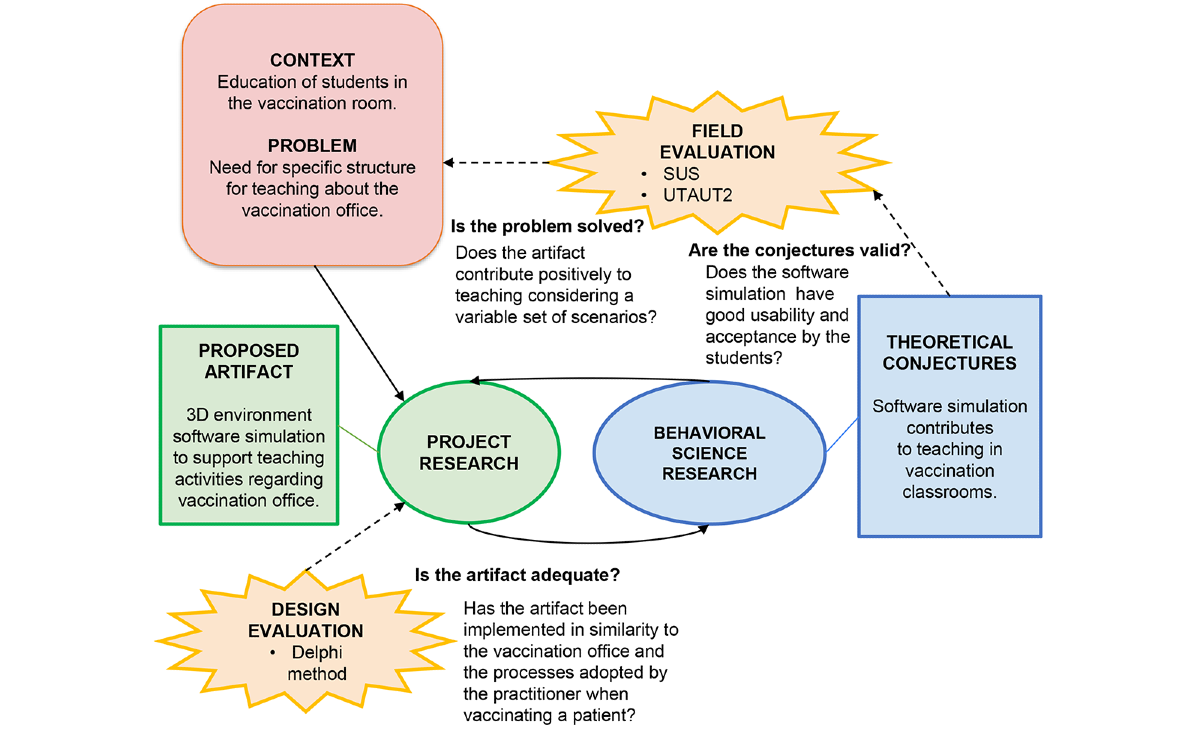
DSR instantiation in this research. DSR: design science research; SUS: System Usability Scale; UTAUT2: Unified Theory of Acceptance and Use of Technology version 2.

The instantiation of the DSR methodology in this research is depicted in Figure 1, following the template proposed by Pimentel et al [10]. Accordingly, its definition follows the 2 main types of research (ie, project research and behavioral science research). The results confront the study’s central hypothesis through both evaluations (ie, design and field). We executed only 1 DSR cycle in this research, which started with project research and concluded with the field evaluation.

### Project Research

Based on the premises presented in the “Introduction” section, the project research objective was to devise a solution, that is, a 3D environment software simulation, to support teaching activities in vaccination rooms. The design and development were performed by including a multidisciplinary team of software and vaccination experts, who cleared the essential requirements and evaluated the design iteratively.

Given the similarity of current simulations with digital games, research about tools used to specify and develop digital games formed the base of this work. Based on relevant and classical models available in the literature, the performed software specification follows the unified model from Hira et al’s work [11] and the educational elements based on Leite and Mendonça [12]. The inapplicable elements to this project (eg, business related) were removed from the model, while educational elements were added. Mock-ups and user interaction diagrams were used to define the virtual vaccination room and the other interface elements.

The simulation was implemented in the Unity game engine (Unity Technologies) due to its popularity and suitability for the intent. The multimedia resources (such as sounds, images, and 3D models) were (Creative Commons 0) licensed or created for the occasion. Furthermore, we developed a desktop system in which the instructor can create and manipulate instances to be executed further on the simulation. The desktop system was developed with the framework Avalonia UI.

The performed evaluation was defined based on previous research [13]. It comprised a design evaluation, still part of the project research, and a field evaluation as part of the behavioral research. Both evaluations used the same software version.

Regarding the design evaluation, a group of experts (with experience in vaccination or related areas, mostly university professors) was invited to compose an expert panel by the end of 2020. The experts evaluated the artifact through the Delphi method [14] to validate its content and general design. Only 1 round was performed in this research, bearing in mind that an expert has regularly assessed the artifact during its creation.

The group tested the 2 simulation scenarios and answered a questionnaire related to the following artifact attributes: objective, structure, presentation, and relevance. For each attribute, the experts classified a set of affirmatives to show their agreement level according to the following status: (1) inadequate, (2) partially adequate, (3) adequate, and (4) fully adequate. Besides, the experts could write down further considerations in a specific space for each attribute or say them aloud during the evaluation.

The consensus achieved by all the experts was measured according to the content validity index (CVI) [15] and the content validity ratio (CVR) [16]. We calculated both variables for each question, the mean of each category, and the mean of the total, considering all questionnaire items.

### Behavioral Research

#### Overview

Behavioral science research seeks an assessment of the solution proposed regarding the hypothesis. The performed evaluation pursued an assessment regarding the software usability and acceptance by students.

#### Scenarios and Questionnaire Sections

We conducted the field evaluation with nursing students, who executed the software simulation running 2 scenarios. Later, the students answered a questionnaire composed of 4 sections: (1) demographic questions; (2) usability-related questions; (3) technology use and acceptance questions; and (4) open questions, in which the participants could express their opinion about the simulation.

The assessment considered 2 typical scenarios. The first scenario depicts the case of a 7-month-old child whose previous vaccines were not administered but are expected in the sixth month of life. The second describes a pregnant woman who needs the Tdap (tetanus, diphtheria, pertussis) vaccine. Both scenarios describe an ordinary real-life situation, demanding apprentices’ analysis of all patient conditions to identify and provide proper care.

As the evaluation objective was not to measure the participants’ knowledge, help was provided as requested. Notwithstanding, we asked the participants to send their performance reports for further analysis. We also considered annotations from the author’s perspective about the experience in the analysis.

The contact with the participants was realized by convenience through email during the first semester of 2021. All the evaluations were executed individually or in groups of up to 3 participants.

Because of the COVID-19 pandemic, the participants interacted through a web conference. Thus, the participants also used their equipment to run the developed software. First, the participants were informed about the research, shown the simulation, and asked to share their computer screens during the experiment to assist when needed.

#### Usability Evaluation

The artifact usability was measured with the System Usability Scale (SUS) [17]. Data collection used a 10-item questionnaire, in which participants must define their concordance level according to a 5-point Likert scale. Following the method assessment calculation, the usability measurement of a given tool/artifact score ranges from 0 to 100. According to Bangor et al [18], the SUS score relates to adjectives, grades, and acceptance ranges.

#### Use and Technology Acceptance Evaluation

**Figure 2:**
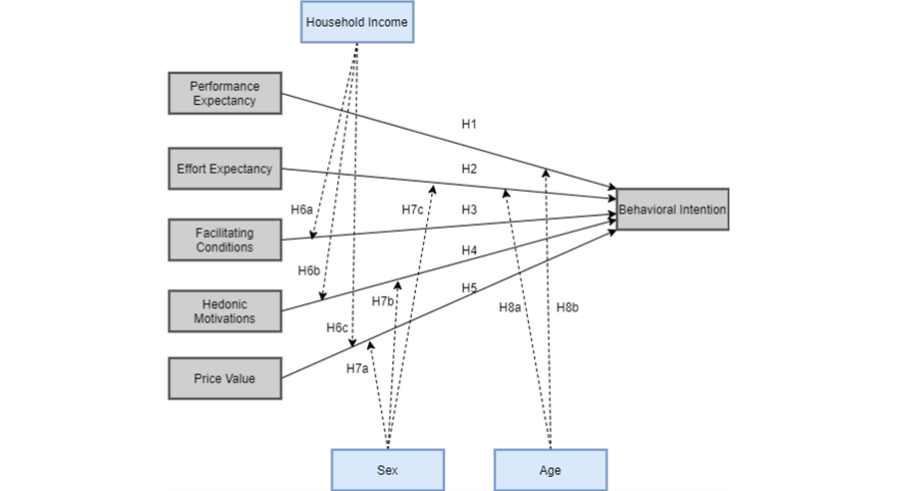
The Unified Theory of Acceptance and Use of Technology version 2 analytical model used in this research.

The Unified Theory of Acceptance and Use of Technology (UTAUT) and its further extension, Unified Theory of Acceptance and Use of Technology version 2 (UTAUT2) [19], are popular tools to analyze the use and acceptance of technology. Those methods were broadly extended and translated into many languages, including a fully adapted Brazilian version [20]. Nishi [20] translated the questionnaire and added a few more moderating variables to the UTAUT2 model. In this work, we used a more suitable version of this modified model (Figure 2).

We removed the constructs “Habit” and “Use Behavior” given the novelty of the artifact. Besides, the “Social Influence” construct was removed because the artifact does not have any social interactions between the participants (and we contacted the participants individually). We also removed the moderating variables “Experience,” “Schooling,” and “Marital status” due to the low variation observed (and expected) in the demographic data. Regarding Price Value (PV), participants considered the artifact under a free software license.

This way, it is possible to analyze the following hypothesis from Figure 2:

- H1 (+): Performance Expectancy (PE) affects the Behavioral Intention (BI) positively;
- H2 (+): Effort Expectancy (EE) affects the BI positively;
- H3 (+): Facilitating Conditions (FC) affects the BI positively;
- H4 (+): Hedonic Motivations (HM) affects the BI positively;
- H5 (+): PV affects the BI positively;
- H6a (+): Household Income (HI) acts as a positive moderating effect on FC;
- H6b (+): HI acts as a positive moderating effect on HM;
- H6c (+): HI acts as a positive moderating effect on PV;
- H7a (–): Sex acts as a negative moderating effect on PV;
- H7b (–): Sex acts as a negative moderating effect on HM;
- H7c (–): Sex acts as a negative moderating effect on EE;
- H8a (–): Age acts as a negative moderating effect on EE;
- H8b (–): Age acts as a negative moderating effect on PE.

We suppose the variable “Age” negatively affects the constructs EE and PE. We believe that young people can learn modern technologies more easily than older people. We also established that the variable “Sex” negatively affects the constructs EE, PV, and HM, knowing that most of the health and welfare course graduates in Brazil (73.8%) are females [21], and this way supports the assessment of its effect on the model. To conclude, we suppose a positive effect of the variable HI on the constructs FC, HM, and PV. This decision assumed that people with higher HI (and thus better social conditions) might be better acquainted with recent technologies. The selection of each hypothesis’s positive or negative effect is related to its interpretation, as displayed in Table 1.

The analytical model used in this research was evaluated using the partial least squares structural modeling equation method and analyzed through the methodology suggested by Benitez et al [22] and Hair et al [23]. We executed a bootstrap process with 100 samples and evaluated each hypothesis according to its relation effect, either positive (ie, supported) or negative (ie, unsupported); only *P* value <5% was considered significant. We developed scripts to automate the data processing, solve the model through the partial least squares path modeling estimation engine SemInR, and report results [24].

**Table 1:** Interpreting the effects of moderating variables.

Variable	Positive effect	Negative effect
Sex	Male	Female
Age	Older	Younger
Household income	Higher	Lower

### Ethics Approval

The evaluation was approved by the Brazilian ethics committee Certificado de Apresentação de Apreciação Ética (CAAE 30545820.2.0000.5151).

## Results

### Overview of Outcomes From the Simulation

The proposed solution is a software simulation suitable for scenario-based training in a standard vaccination room modeled as a 3D environment. In this environment, a nurse avatar in a typical workday represents the user apprentice. A typical scenario assumes all required procedures before general servicing was performed, including preparing and opening the vaccination room.

**Figure 3:**
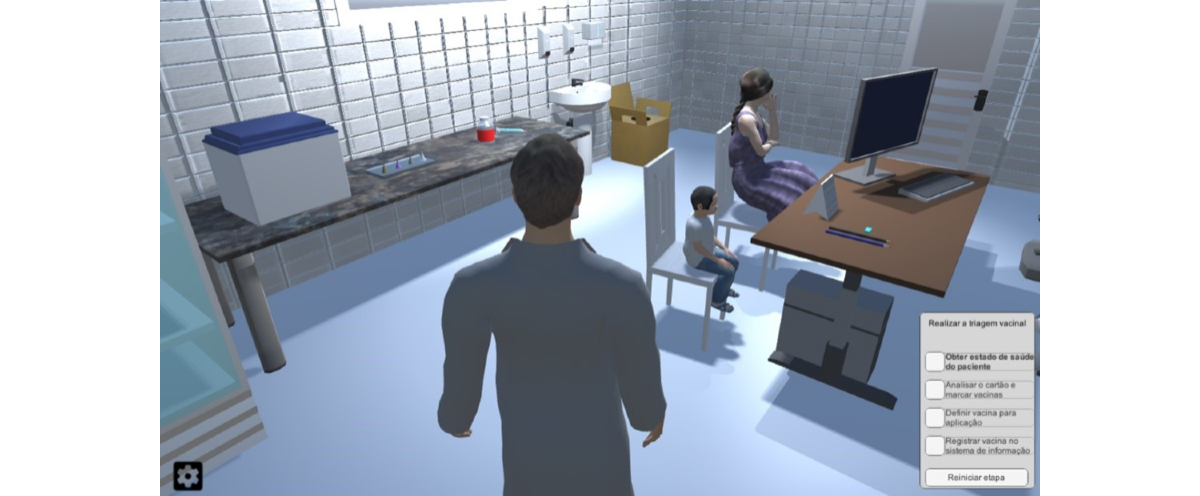
Starting view of the simulation environment (i.e., vaccination room).

Upon starting the simulation, apprentices must move their avatar toward the virtual patient in the room. Figure 3 illustrates the apprentice’s view when starting the simulation in a scenario with a mother and her child. The avatar moves through the room when pressing the keys W, A, S, and D (or the keyboard arrows), and mouse clicks provide interaction with some elements in the environment.

The tasks emulate the real-life process, except for checking the postvaccination adverse event (PVAE). While checking the PVAE, the nurse should observe whether the patient shows any immediate unexpected reaction. In the positive case, the nurse needs to react to them accordingly. We decided not to implement it because each event can be unique and vary for each patient, thus representing a higher complexity to replicate in the simulation.

Thus, the stages and respective tasks to be performed by the apprentice in the simulation are follows:

- Vaccine screening: analyze the patients’ health conditions, analyze their vaccination card, define the vaccines to be administrated, and register the vaccines in the information system.
- Hand hygiene: use the liquid soap dispenser, the paper towel, and the alcohol-based hand disinfectant dispenser.
- Preparation of the vaccines: select the vaccine administration route, select the needle size and dose, and remove the vaccine from the thermal box.
- Vaccination: apply the vaccine and dispose of the materials in the correct bin.
- Process finalization: set the return date and tell the patient to leave.

The simulation allows scenarios with the possibility of applying more than 1 vaccine. In this case, the apprentice repeats stages 3 and 4 until the administration of all vaccines.

The virtual room is divided into 3 sectors to consolidate the interaction style. The sectors gather a collection of related elements in a similar context: screening, hand hygiene, and vaccine preparation. As the apprentice selects 1 of these sectors, the camera view changes to a fixed position from the sector. Thus, the apprentice can interact with all needed elements and execute tasks properly.

Multimedia Appendix 1 presents a comprehensive report with many screenshots and detailed descriptions of the proposed software simulation.

The thermal box contains most vaccines presented in the Brazilian 2020 schedule. Thus, in a standard scenario, the apprentice must select a vaccine from 18 options (Multimedia Appendix 1). The vaccines presented by default are available through typical (ie, actual/ordinary) scenarios. However, an instructor can set up new vaccines in custom scenarios, allowing simulation considering vaccines with unique characteristics.

The software actively records user actions through the simulation process, in which the user can export the respective report as an external file. Considering that an incorrect action prevents the simulation flow, recorded as a wrong choice, it is worth mentioning that all vaccine combinations are accepted as a valid input. As the correct administration varies according to each vaccine requirement, considering the patient’s age, muscle state, and even unique medical conditions, some scenarios might demand a detailed assessment of the report data.

The educational assessment is related to how well the apprentice executed the vaccination process, considering the number of incorrect interactions. Thus, when concluding the simulation, the performance report displays the following items:

- The last stage performed.
- List of vaccines administered.
- List of vaccines to be administered in the future as per the patient vaccines card.
- The number of incorrect selections or inputs when:
  Defining a vaccine to be administrated.
  Defining a return date.
  Interacting with the information system.
  Interacting in the hygiene sector.
  Defining the administration route (total and specific by vaccine).
  Defining the needle size (total and specific by vaccine).
  Defining the dose (total and specific by vaccine).
  Selecting a vaccine flask from the thermal box (total and specific by vaccine).

### Auxiliary Module for Instructors—Scenario Management

The auxiliary system allows the instructors to specify and manage parameters to create (or edit) a simulation scenario for their students. This system also aids in examining the performance report. The scenario variable options, organized into 3 categories, are presented in Textbox 1.

**Figure 4:**
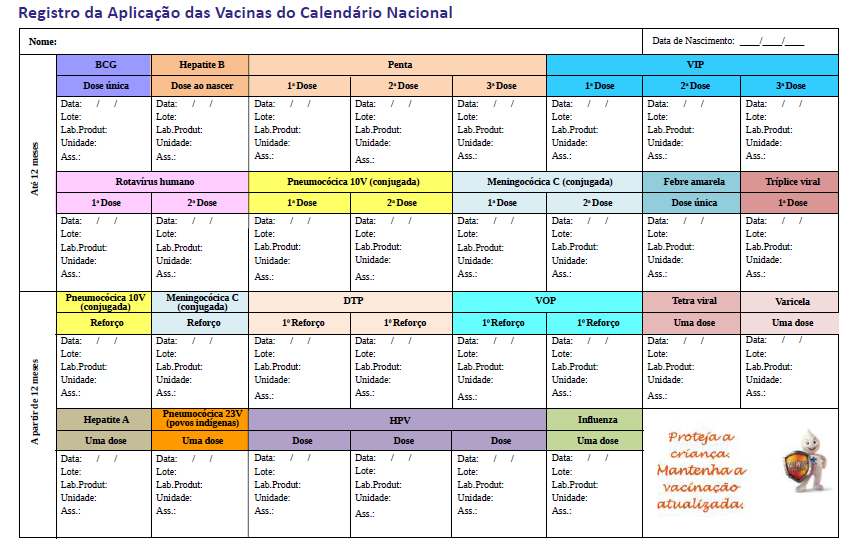
Current vaccination card (first page) used nowadays in Brazil.

**Figure 5:**
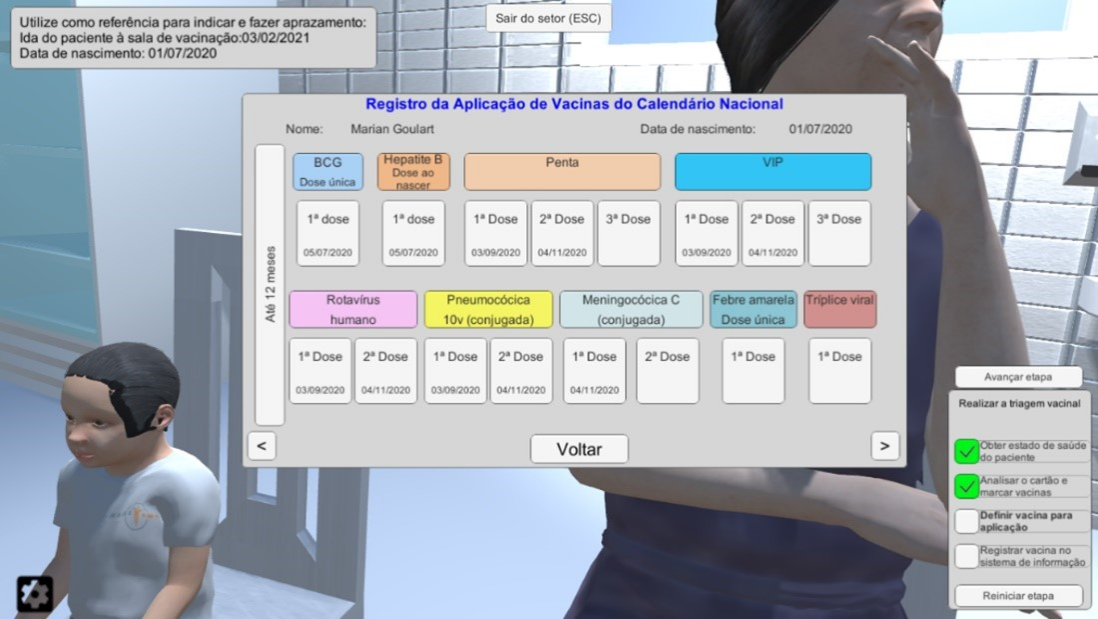
Implementation of the current vaccination card in the simulation (first page).

The system provides 7 avatars (ie, 3D character models) and 5 different vaccination cards to grant more realism and educational possibilities. Besides the current vaccination card, the system provides 4 other card models previously used in Brazil. Figures 4 and 5 show the current vaccination card: the real-life card and the implemented version in the simulation, respectively.

**Scenario variable options for simulation.**

*Basic parameters*

- Patient’s name
- Patient’s birth date
- Patient’s 3D model
- Companion’s 3D model
- Scenario description
- Consultation date
- Opening dialog text

*Patient’s health conditions*

- Preexisting diseases and allergies
- Medication being used
- Reactions to previous vaccine administrations

*Vaccination history*

- Type of vaccination card
- Expected return date
- Vaccines administrated previously
- List of possible vaccines to be administrated by the apprentice
- Permission to use special fields in the vaccination card (the apprentice can set any vaccine in this field)
- Permission to apply a vaccine that is not listed in the current schedule

### Design Evaluation

A total of 9 experts participated in the evaluation. The age of 5 experts is between 30 and 39 years, 1 between 40 and 49 years, and 3 between 50 and 59 years. All experts have graduated in nursing; besides, one holds a master’s degree and 8 a PhD. Table 2 presents their overall professional experience in nursing. Further, we noted 5 specialization areas and all experts work as professors in 4 Brazilian universities.

Table 3 summarizes the experts’ answers regarding the artifact’s objectives, structure, presentation, and relevance. According to the number of participants, the expected CVR to approve an item is 0.78, whereas the CVI rates need to be higher than 75% [15].

Some experts pointed out that the hand hygiene process was inadequate, mainly because the alcohol and liquid soap dispensers were displayed swapped according to new recommendations. Besides, some simulation elements displayed poor representation, such as the syringe and the flask used to represent the dose selection, and the avatars’ 3D models that were not fully matching their description.

One expert also mentioned that the error messages were confusing and occasionally could not recognize their cause. To conclude, many participants took a while to grasp the “Advance Stage” button—used to advance the vaccine definition task after the user selected all vaccines to be administrated—and suggested changing its place on the screen.

Regarding their performance reports, Tables 4 and 5 summarize the results. Notably, the experts struggled more with the hand hygiene interaction, selecting a vaccine flask from the thermal box, and in the needle selection task. The return date was inserted incorrectly by 1 expert. Besides, the remarkable difference between the number of errors caught in both scenarios suggests that the experience acquired in the first scenario resulted in fewer interaction errors in the following scenario. Finally, 2 experts did not send their reports, while E6 and E7 performed only 1 scenario.

**Table 2:** Experts’ characterization according to their professional experience.

Years since graduation	Number of experts
0-9	2
10-19	2
20-29	4
30 or more	1

**Table 3:** Results of the Delphi questionnaire for the design evaluation.

Attribute/item			Range	SD			Content validity ratio		Content validity index		
**Objectives**											
	1. The information/content is consistent with the educational needs of the target audience (undergraduate students).	3-4			0.31	1		100			
	2. The information/content is important for the quality of vaccination education.	3-4			0.31	1		100			
	3. It invites or instigates changes in the behavior and attitude of the students (future professionals).	2-4			0.67	0.78		89.88			
	4. It can be circulated in the scientific/educational environment of the nursing field.	3-4			0.42	1		100			
	5. It meets the objectives of undergraduate nursing courses.	4-4			0	1		100			
**Structure and presentation**											
	1. The material looks attractive.	3-4			0.50	1		100			
	2. The content is adequate.	3-4			0.42	1		100			
	3. The information presented is scientifically correct.	2-4			0.67	0.78		88.89			
	4. There is a logical sequence of the proposed content.	3-4			0.48	1		100			
	5. The information is well structured in concordance and spelling.	3-4			0.31	1		100			
	6. The writing style corresponds to the level of knowledge of the target audience.	4-4			0	1		100			
	7. The illustrations are expressive enough.	3-4			0.47	1		100			
**Relevance**											
	1. The themes reinforce aspects that should be reinforced.	4-4			0	1		100			
	2. The material covers the subjects needed for vaccination knowledge.	4-4			0	1		100			
	3. It proposes the construction of knowledge.	4-4			0	1		100			
	4. The material allows the transfer and generalization of vaccination learning.	3-4			0.42	1		100			
	5. It is suitable for use in teaching vaccination.	3-4			0.31	1		100			

**Table 4:** Interaction errors (7-month-old child) present in the experts’ performance reports.

Items	Participant							Total
	E1^a^	E2	E3	E4	E5	E6		
Interacting in the hygiene sector	0	0	3	8	4	5	20	
Set a wrong vaccine in the card	0	0	0	0	0	0	0	
Interacting with the computer (ie, information system)	1	2	5	0	1	0	9	
Select the administration route	0	0	0	0	0	0	0	
Select the needle	0	0	0	17	1	0	18	
Select the dose	0	0	2	0	0	2	4	
Select a vaccine flask from the thermal box	2	0	1	17	1	1	22	
Defining a return date	1	0	0	0	0	0	1	

^a^E: expert.

**Table 5:** Interaction errors (pregnant woman scenario) present in the experts’ performance reports.

Items	Participant							Total
	E1^a^	E2	E3	E4	E5	E7		
Interacting in the hygiene sector	0	0	0	0	3	0	3	
Set a wrong vaccine in the card	0	0	0	0	0	0	0	
Interacting with the computer (ie, information system)	0	0	0	0	0	0	0	
Select the administration route	0	0	0	0	0	0	0	
Select the needle	0	0	0	0	0	2	2	
Select the dose	0	0	0	0	0	0	0	
Select a vaccine flask from the thermal box	0	0	0	0	0	2	2	
Defining a return date	0	0	1	0	0	0	1	

^a^E: expert.

### Field Evaluation

The field evaluation was conducted with 20 participants (14 females and 6 males). All participants study in the Universidade Federal de São João del-Rei (undergraduate or graduate students); 7 are nurses. They are all less than 30 years of age, with the majority being single; however, 1 was married and 1 had a common-law marriage relation. The number of students with HI grouped by the Brazilian minimum salary (BMS; around US $200/month) was as follows: 5 with income below 2 BMS, 9 with income over 2 BMS and below 4 BMS, and 9 over 4 and below 10 BMS. Only 3 participants related a previous experience with virtual reality applications, games, or similar.

The overall SUS score obtained from the mean of all students’ scores was 81.4. Thus, the software simulation usability is acceptable, associated with the adjective *good* and grade B.

Table 6 presents the descriptive statistics of the UTAUT2 questionnaire: the mean (SD) and the range for each question.

Multimedia Appendix 2 presents the measurement model evaluation results, in which all observed values comply with the standard criteria. Therefore, this assessment grants internal data validity to the measurement model.

The discriminant was validated by the Fornell-Larcker criterion [25]. Establishing discriminant validity implies that a construct is truly distinct from others by empirical standards and captures phenomena not represented by other constructs in the model. The results, available in Multimedia Appendix 2, show a valid relation when the value in the main diagonal of the table is greater than any other in the same column. Thus, the discriminant validity was achieved by all relations between the constructs, except for FC and EE. Nevertheless, we established the model for this research according to the UTAUT2 analytical model.

According to the structural model evaluation results (Table 7), we can only state hypothesis H8a as supported. The other results from the structural model evaluation, showing the indirect effects and the constructs’ *R*^2^ value, are provided in Multimedia Appendix 2.

Table 8 shows the results of the 15 submitted performance reports. The highest number of errors in the field simulation occurred when interacting in the hygiene sector, selecting a vaccine flask from the thermal box, and interacting with the computer (ie, simulated health information system).

Finally, the answers to open questions about the simulation’s positive and negative points and suggestions or critics about it and the whole experiment are summarized in Textbox 2.

**Table 6:** Descriptive statistics of the UTAUT2^a^ questionnaire.

Item	Mean (SD)	Range
PE1^b^: I find the tool useful in my daily life.	6.55 (0.60)	5-7
PE2: Using the tool increases my chances of achieving things that are important to me.	6.30 (1.03)	3-7
PE3: Using the tool helps me to accomplish things more quickly.	6.10 (0.79)	5-7
PE4: Using the tool increases my productivity.	6.20 (0.83)	5-7
EE1^c^: I find the tool easy to use.	6.20 (1.26)	3-7
EE2: It is easy for me to become skillful at using the tool.	6.50 (1)	3-7
EE3: My interaction with the tool is clear and understandable.	6.30 (0.92)	4-7
EE4: Learning how to use the tool is easy for me.	6.45 (0.83)	5-7
FC1^d^: The tool is compatible with other technologies I use.	6.25 (1.20)	4-7
FC2: I have the resources necessary to use the tool.	6.35 (0.99)	4-7
FC3: I can get help from others when I have difficulties using the tool.	6 (1.29)	2-7
FC4: I have the knowledge necessary to use the tool.	6.35 (0.93)	4-7
HM1^e^: Using the tool is fun.	6.60 (0.60)	5-7
HM2: Using the tool is enjoyable.	6.45 (0.68)	5-7
HM3: Using the tool is very entertaining.	6.25 (0.97)	4-7
PV1^f^: The tool is reasonably priced.	6.45 (1.10)	4-7
PV2: The tool is a good value for the money.	6.55 (0.89)	4-7
PV3: At the current price, the tool provides a good value.	6.40 (1.10)	4-7
BI1^g^: I intend to continue using the tool in the future.	6.35 (0.99)	4-7
BI2: I will always try to use the tool in my daily life.	5.75 (1.16)	3-7
BI3: I plan to continue to use the tool frequently.	5.65 (1.35)	3-7

^a^UTAUT2: Unified Theory of Acceptance and Use of Technology version 2.

^b^PE: Performance Expectancy.

^c^EE: Effort Expectancy.

^d^FC: Facilitating Conditions.

^e^HM: Hedonic Motivations.

^f^PV: Price Value.

^g^BI: Behavioral Intention.

**Table 7:** Total direct effects and hypothesis validation.

Hypothesis	Relation	Total direct effects	*P* value
H1	PE → BI	0.593	.58
H2	EE → BI	0.549	.74
H3	FC → BI	–0.356	.60
H4	HM → BI	–0.321	.42
H5	PV → BI	0.489	.85
H6a	HI → FC	–0.024	.91
H6b	HI → HM	–0.136	.54
H6c	HI → PV	0.100	.62
H7a	Sex → PV	0.297	.34
H7b	Sex → HM	0.273	.57
H7c	Sex → EE	0.320	.27
H8a	Age → EE	–0.385	.05
H8b	Age → PE	–0.322	.19

**Table 8:** Interaction errors caught in the field evaluation.

Item	Total, n
Interacting in the hygiene sector	24
Set a wrong vaccine in the card	0
Interacting with the computer	11
Select the administration route	0
Select the needle	5
Select the dose	6
Select a vaccine flask from the thermal box	19
Defining a return date	3

**Positive and negative points regarding the simulation.**

*Positive points*

1. Ease of use.
2. It is fun (ludic element).
3. The tool has proximity to the reality and experience in the vaccination room, according to the safety rules of the Brazilian National Program of Immunizations.
4. It allows the user to visualize situations that minimize errors.
5. It allows the user to remember the vaccination process step-by-step—1 participant emphasized the hand hygiene process.
6. Easy to learn how to use.

*Negative points*

1. The avatar and the camera system are challenging to handle.
2. The interaction with some aspects of the vaccination room is tricky—mainly with items from the hygiene sector.
3. The patient interaction in the scenario with a child, not the adult, negatively affects realism.
4. The patient does not move to the stretcher to receive the vaccine administration, which negatively affects realism.
5. Because of a lack of knowledge in the use of computers, learning how to use the simulation is challenging.
6. Equipment with high processing power is required because 1 male participant had difficulties in using the simulation with his equipment.

## Discussion

### Principal Findings

This paper presents a novel software-based simulation providing a 3D environment where an apprentice must complete the vaccination process according to a variable set of scenarios and Brazilian standards. An additional module was also developed to manage simulation scenarios and view performance reports. The creation considered the identified problem and opportunities along with its development, following the DSR method. The main results and in-depth discussion regarding the evaluations are presented in the following sections.

The proposed simulation was created to support teaching in the vaccination room, using innovative methods and technological resources. The design assessment concluded with activities regarding the project research, while the behavioral science research concluded with field evaluation. Thus, we conducted 2 evaluations to answer the research questions related to the project research (ie, design evaluation) and the behavioral research (ie, field evaluation).

Regarding the design evaluation, the experts considered the artifact approved according to the results of the design evaluation. The artifact modeling and its implementation are adequate (with possible minor improvements, such as a better 3D representation of some objects, inclusion of the PVAE’s procedure, and the information screen). The experts stated its high relevance to teaching and learning. That was seen not only in the “Relevance” attribute but also in the qualitative feedback.

In the field evaluation, nursing students assessed the artifact’s relevance. This evaluation aimed to evaluate the simulation’s usability and its use and acceptance by the students (and potential future users).

Usability was evaluated using the SUS. The results indicate a final score of 81.4 points, and an acceptable usability grade, which also can be described as good.

The students also indicated a remarkable acceptance level of the technology through the UTAUT2-based evaluation. PE was the factor that most influenced the students’ BI to continue using the technology. The simulation was easy to learn, and the SUS final score was reflected by the opinions of various participants (students and experts).

Both evaluations pointed at the ease of use and learning as positive aspects, although some participants struggled with the interaction system. The main difficulties reported were moving the avatar through the virtual environment, accessing the hand hygiene sector, and understanding the task list system. Remarkably, all participants completed the virtual vaccination process during the experiment.

### Design Evaluation

The results of the “Objectives” attribute (Table 3) allow us to infer that the simulation meets its primary goal. The content and information displayed in the simulation meet the goals of nursing courses (CVR=1; SD 0). Moreover, the content and the information displayed in the simulation are coherent with the target audience (CVR=1; SD 0.32). Still, except for item 5, not all experts present the same agreement level, given the SD variation identified and the qualitative results.

Specifically, item 3 achieved the minimum acceptable value according to the CVR criterion. The reason may be related to the hand hygiene process, with the dispensers’ position switched, as pointed out by some experts. They also noted an analogous situation in the “the information presented is scientifically correct” item from the “Structure and Presentation” section.

One expert said that the hand hygiene process was inaccurate regarding the most recent recommendation. A nurse may prefer washing hands with liquid soap rather than alcohol gel, but the 2 products should not be used simultaneously according to the most recent standard recommendation. In addition, the expert proposed the removal of the sink from the simulation, allowing only 1 way of washing hands.

However, it is worth mentioning that using both products together is not a bad practice in all cases. Besides, there are circumstances where a nurse cannot clean their hands with either soap or alcohol. To sum up, the professional must judge the situation and choose the best alternative. Thus, we dropped the suggestion of removing the sink and changing the hand hygiene sector.

We decided not to implement the PVAE due to its idiosyncrasies. Nevertheless, it is a required step. Many experts suggested adding at least a reminder at the end of the process as an educational feature.

The “Structure and Presentation” assessment shows that the writing style corresponds to the target audience’s knowledge. No problems related to grammar and spelling were noticeable in the simulation, and it uses the specific technical terms appropriately. The experts approved the simulation’s visual quality, and the variation between the agreement levels (adequate and fully adequate) is related to the qualitative feedback.

Because of the experts’ possible lack of experience in computer applications (such as digital games or simulations) and the adopted interaction style, we expected hurdles concerning recognition and interaction within the 3D simulation environment. The performed assessment reflects it through several items. It is worth mentioning that the interaction style applied in the proposed software simulation is a well-consolidated pattern in the software industry, which is present in many modern digital games.

Some experts struggled to move the avatar, which also handicapped their interaction with particular objects in the environment. When the interaction context changes—from the avatar view to a given sector view—selecting items from that sector can be impossible due to an overlay of the avatar mesh with the sector objects. The most prominent errors observed during the experiments in the hand hygiene sector (Tables 4 and 5) are related to this interaction issue.

Several experts tried to set the patient’s return date on the computer instead of initially interacting with the virtual patient. According to the real-life procedure, a nurse confirms the returning date calculated by the standard information system, as long as the patient updates the vaccination schedule. Otherwise, the nurse must input the new return date directly into the system based on the patient’s vaccination card.

We chose to set the return date at the end of the simulation through interaction with the patient. The reason was the high complexity involved in replicating the automatic process executed by the standard information system in the simulation. However, it can indicate a simulation mistake because the current metaphor drifts apart from real life. It is also possible to insert the return date directly into the patient’s vaccination card. Still, it was not a required step, and only 1 participant used this option during the field and design evaluations.

Some experts tried to register the vaccine in the system more than once. That happened because of an initial misunderstanding about stage transition and the overall task list comprehension.

Despite minor interaction problems observed and few experts’ remarks to add a higher degree of realism and trustworthiness to the simulation, the whole artifact was approved. The final mean of both CVI and CVR was above the expected value.

### Field Evaluation

The usability assessment obtained from the SUS score substantiates the participants’ opinions regarding ease of use. Among other positive points mentioned by the students, the ludic learning factor and artifact relevance were also highlighted.

Furthermore, according to the participants, “the tool presents similarity with the PNI safe practices,” whereas the professionals noted that “use matches the reality lived in the vaccination room.” Thus, it is an adequate tool to support teaching and learning experiences. The user “has the vision of how it is like to be in a vaccination room,” it allows to “practice before interacting with a real patient,” and thus the simulations allow to “visualize situations that diminish missteps.”

Similar to the situation with some experts, some students also struggled to handle the avatar movement and interaction with other elements. The interaction style defined for the movement is standard in the digital games industry. Nevertheless, it is not a natural interaction style for all. Unexperienced users need directions and time to properly learn how to handle the avatar.

The errors related to computer interaction can be explained by the lack of comprehension regarding the changing stages through the simulation. As observed in the design evaluation, the students also tried to register the vaccine on the computer more than once. One student attempted to write the return date as well. The reasons are the same as those discussed in the “Design Evaluation” section.

Students also clicked more than once in vaccine flasks from the thermal box, and a few just unthinkingly clicked on random vaccines. The interaction with the thermal box was perceived as simple to be achieved and understood. Yet, the lack of attention or experience with similar software increases the number of errors.

Whereas the previous mistakes are understandable and tamed mainly by experience, those in the hand hygiene sector indicate a significant issue. Only 4 students from the 15 who sent the report did not record missteps in this area. This sector was also extensively discussed in the experts’ evaluation.

Regarding the UTAUT2 evaluation, we can only generally support hypothesis H8a due to the statistical significance (Table 7). The conclusion is that being younger has a positive influence on the construct EE.

The UTAUT2 results are complementary and supported by the SUS score and qualitative feedback. Although the UTAUT2 method is unsuitable for a small number of participants because it may lack statistical reliability, it is valuable as a theoretical framework to guide technology assessment and provide relevant results.

Fully achieving statistical significance was not our goal nor the expected result, bearing in mind the limited number of participants we could gather. Thus, despite its limitations, the results are still valid and represent the participants’ perceptions well. Consequently, UTAUT2 is used as a theoretical framework to understand the simulation through the participants’ view and not as a final and general assessment.

Therefore, we noted that the PE positively affects the BI of the students in continuing to use the simulation (H1). In this case, the users feel compelled to continue using the simulation because they perceive its benefits. Regarding the scenario performance, all students completed the vaccination process and pointed out most factors as positive regarding their experience. Their performance is also reflected in Table 8, in which their number of missteps is remarkably smaller than that of the experts’ (Tables 4 and 5).

EE also contributes positively to BI (H2), according to the participants. Thus, the simulation’s challenge is adequate for the audience. Although few experts had the impression of a lack of logical sequence for the simulation’s tasks, the arrangement was perceived as positive from the students’ perspective. Users need to comprehend the environment and the task to accomplish its purpose by moving the avatar and interacting with the suitable element. This process presents a reasonable challenge and resembles the real-life process. As 1 male participant noted in his qualitative feedback, the simulation tool is close to the reality and experience in vaccination rooms.

By contrast, the influence of FC diminishes the user’s BI (H3). Few students complained about the lack of knowledge, experience, and training regarding computer systems, besides a steep learning curve associated with the simulation interaction procedure. Few experts also noted the lack of clear instructions in the simulation, which can be a problem. However, the learning needs seem not to be an issue because the struggling participants could use the simulation with only few initial instructions. Moreover, the FC construct assessment achieved a high score, and it is worth noting the overall high SUS score and the high EE and PE values.

The HM also does not confer positive effects in students’ BI (H4). The artifact was built according to simulation concepts but not as a game, and some students also emphasized it. However, we expected a positive influence considering the target audience (young adults and students aged between 19 and 27 years) and the similarity of the artifact with a digital game. Nonetheless, some students mentioned that the simulation was fun as a positive aspect.

The PV contributes positively to the BI (H5). The participants considered the artifact available under a free software license. Thus, the artifact was recognized as having a good cost-benefit because it is free and benefits the participants.

Regarding the influence of sex, hypotheses H7a, H7b, and H7c positively affected the constructs PV, HM, EE, and BI. Accordingly, female participants are less influenced by the characteristics of price, HM, and effort expectation. Furthermore, sex directly affects the students’ BI of the continued use of the artifact, with men being more favorable than women.

Regarding the variable age, we can note that it has adverse effects on EE (H8a) and PE (H8b) and on BI according to our sample. This finding indicates that younger users have better effort and performance expectations regarding simulation use. It matches our observation regarding the experiment and the discrepancy in the number of errors from experts and students. According to our sample, the *R*^2^ value indicates that the variable age explains 10.3% of the PE variance. In association, 19.4% of the EE variance is explained through age and sex. Besides, hypothesis H8a was supported and achieved statistical significance in our model. Therefore, younger users have higher BI in using the simulation than older ones.

To conclude the structural model analysis, all other constructs predict the users’ BI on the continued use of the artifact in 73.3% of the cases, according to the *R*^2^ value in our sample. Moreover, the analysis shows the influence of variable age with statistical significance favorable toward young users on the EE. PE, EE, and PV are the influential positive factors of the BI in the structural model built. Although the PV (ie, artifact considered free software) affects BI, HI is neglectable.

The BI1, BI2, and BI3 items from Table 6 assess the participants’ BI, reaching an overall average of 5.92. Despite the smaller score compared with other constructs, the general intention is optimistic. Although some participants are neutral, most declare the intention to continue using the simulation (BI1). Most students will also try to use the tool as much as possible (BI2). The same argument can be extended to item BI3, in which the students intend to use the simulation frequently. The low SD compared with the average indicates a high agreement level.

Although there is still considerable debate regarding the potential usefulness of serious games, previous research shows that such digital approaches appear to be at least as effective as controls and, in many studies, more effective for improving knowledge, skills, and satisfaction [26]. Further rigorous and theory-driven research is required and could promote better understanding, leading to enhanced design processes and outcomes.

Both evaluation audiences cited the high relevance of the proposed simulation for teaching. Points for improvement were also reported.

### Strengths and Limitations

The lack of end users in the design process is a notable limitation concerning the collaborative co-design team, but the evaluations revealed their perceptions and points for improvement. The evaluations were conducted entirely online, using participants’ computers. Students required special assistance and sometimes demanded brief interventions during the experiment. Before the evaluation stage, a brief introduction about the interaction with the simulation may have added a bias to the participants’ answers. Besides, participants had different assumptions about simulation performance because each used a different computer.

Regarding the UTAUT2 evaluation, the sample size is also seen as a limitation of this study. Although PLS-PM has many advantages over other methods, being more reliable when the sample size is small [23], its performed analysis is limited. According to Hair et al [27], each construct demands at least five participants, while 15-20 will be ideal, yet without convergence assurance. Notwithstanding, the method and the sample allowed a limited but still solid analysis.

Nonetheless, the model assessment granted internal validity to all criteria and mostly achieved the discriminant validity. The discriminant validity was not established between the constructs FC and EE, but an analogous situation was reported in the UTAUT2 model proposition [19]. Further, the model has not achieved statistical significance in almost all the hypotheses. However, the model was kept as theoretically proposed because the results obtained from the other evaluations complement and support the results found in the UTAUT2 analysis. It is still important to mention that the results were validated by a statistic professional who also assisted in the initial analysis of the results to ensure higher reliability for the study. Moreover, results obtained from the other evaluations complement and support the results found in the UTAUT2 analysis.

### Conclusions

The research accomplished its goal of creating a software-based simulation to support teaching scenarios in the vaccination room. The evaluation results showed that the proposed simulation is adequate, with good usability and student acceptance.

The design evaluation indicates that the artifact allows transferring, sharing, and generalizing the knowledge. Therefore, the created simulation is suitable to be used in vaccination education.

Given all the assessed elements that influence the users’ BI and the qualitative feedback provided, students approved the artifact: the simulation presents good usability, and its users accept it well.

From the participants’ point of view, the simulation had a more significant focus on the educational experience. At the same time, HM was seen as a secondary element. This result meets our theoretical foundations in simulations and digital games. However, a positive influence was expected given the target audience of this evaluation: young adults. Both HM and FC are detractor factors to the BI.

We propose as future work adding different vaccination schedules to be selected by the instructor and exploring the simulation with other tasks, such as opening the vaccination room and those related to the vaccine conservation. Besides, it is necessary to validate the UTAUT2 with more participants to validate the entire model used in this study.

## Appendix

Multimedia Appendix 1 Details of the proposed software simulation.

Multimedia Appendix 2 Details of the use and technology acceptance evaluation.

## Abbreviations

BI: Behavioral Intention
BMS: Brazilian minimum salary
CVI: content validity index
CVR: content validity ratio
DSR: design science research
EE: Effort Expectancy
FC: Facilitating Conditions
HI: Household Income
HM: Hedonic Motivations
PE: Performance Expectancy
PNI: National Program of Immunizations (in Brazil)
PV: Price Value
PVAE: postvaccination adverse event
SUS: System Usability Scale
UTAUT2: Unified Theory of Acceptance and Use of Technology version 2

## Data Availability

The data sets generated during or analyzed during this study are not publicly available due to compliance with ethical standards on research with humans but are available from the corresponding author on reasonable request.
