# Supplementary material for: Software-Based Simulation on a 3D Environment for Vaccination Teaching and Learning: Design Science Research
Source: JMIR Med Educ. 2022 Dec 2;8(4):e35712. doi: 10.2196/35712 (PMC9758638; doi:10.2196/35712)
Supplement: Multimedia Appendix 1 [file mededu_v8i4e35712_app1.pdf]

Multimedia Appendix 1

Details of the proposed software simulation

The application starts with a menu screen showing three options, as shown in Figure 1. To start a new simulation, the user must click on the button "Nova simulação" ("New simulation"). Clicking on the button "Informações" ("Information"), the program displays an image showing the simulation interaction scheme. Simulation options can be set by clicking on the button "Opções" ("Options"). The configurations allow the user to set the system folder with the scenario files and the interaction element highlighting feature in the 3D environment. Starting a simulation, the user apprentice must select a scenario instance, following the simulation mode (i.e., training or test).

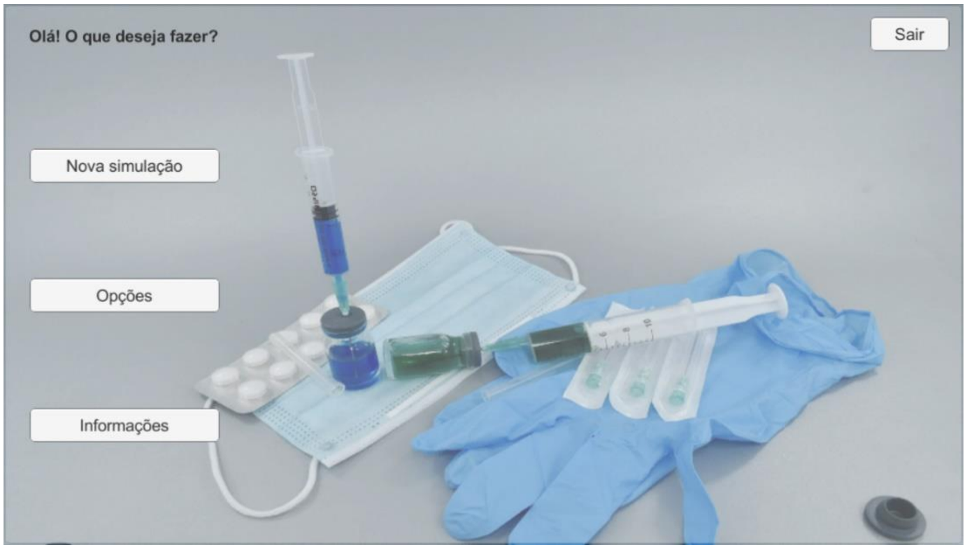

Figure 1: Simulation main menu screen.

Starting the simulation, the apprentice must move its avatar towards the virtual patient in the room. Figure 2 illustrates the apprentice's view when starting the simulation in a scenario with a mother and her child. The avatar moves through the room by pressing keys W, A, S, and D (or the keyboard arrows), and mouse clicks provide interaction with some elements in the environment.

The focus of the simulation is on the vaccination process performed by the apprentice. The task list shown at the right bottom of the screen (Figure 2) is updated accordingly to the process completion. The task list is not visible when the simulation is in test mode.

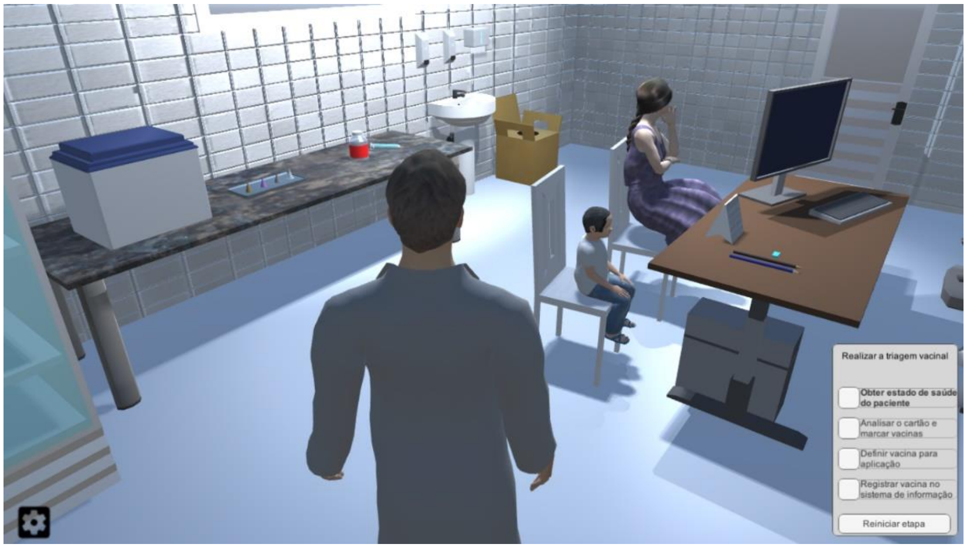

Figure 2: Starting view of the simulation environment (i.e., vaccination room).

The tasks emulate the real-life process, except for checking the adverse events following immunization (AEFI). While checking the AEFI, the nurse should observe if the patient shows any immediate unexpected reactions. In the positive case, the nurse needs to react to them accordingly. We decided not to implement it since each event can be unique and vary for each patient, thus representing a higher complexity to replicate in the simulation.

Thus, the stages and respective tasks to be performed by the apprentice in the simulation are:

1. Vaccine screening: analyze the patient's health conditions, analyze his vaccination card, define the vaccines to be administrated and register the vaccines in the information system.
2. Hand hygiene: use the liquid soap dispenser, the paper towel, and the alcohol-based hand disinfectant dispenser.
3. Preparation of the vaccines: select the vaccine administration route, select the needle size and dose, and remove the vaccine from the thermal box.
4. Vaccination: apply the vaccine and dispose of the materials in the proper box.
5. Process finalization: set the return date and tell the patient to leave.

The simulation allows scenarios with the possibility of applying more than one vaccine. In this case, the apprentice repeats Stages 3 and 4 until the administration of all vaccines.

The interaction with the simulation elements occurs through the mouse's left-click. A highlight interaction system, where hovering the mouse pointer over an element changes its color to gray to indicate possible interaction, was implemented to assist users during the simulation. It also describes the action at the bottom of the screen (Figure 3).

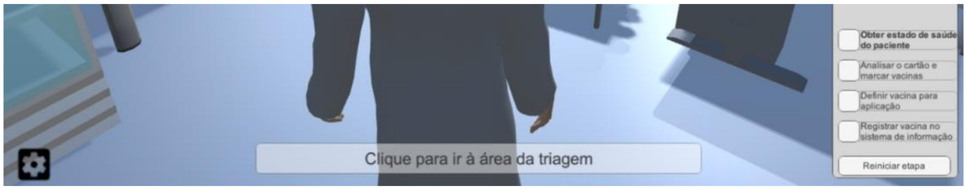

Figure 3: The highlight interaction system emphasizes elements turning them gray and displays the action description at the bottom of the screen – Click to go to the screening desk.

Along with the highlight system, we divided the room into three sectors to consolidate the interaction style. The sectors gather a collection of related elements in a similar context: screening, hand hygiene, and vaccine preparation. As the apprentice selects one of these sectors, the camera view changes to a fixed position from the sector – as illustrated in Figure 4, Figure 5, and Figure 6. Thus, the apprentice can interact with all needed elements and execute tasks properly.

In the screening sector (Figure 4), the camera is set behind the table. The apprentice has access to the patient, computer, and calendar. The interaction with patients consists of several dialog options to gather information to provide proper care - define required vaccines and return dates. As the regulation requires, the apprentice must register the vaccines figuratively in an information system when interacting with the computer.

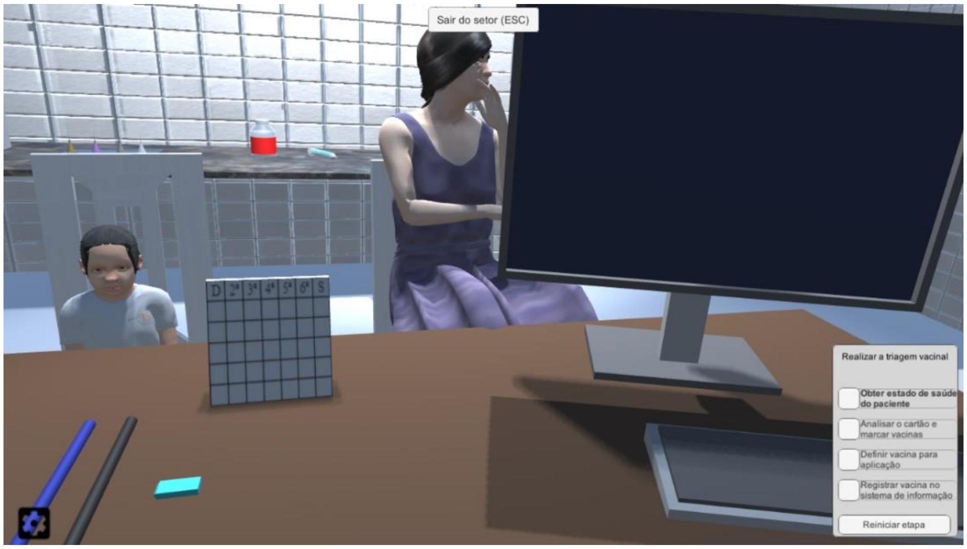

Figure 4: Vaccine screening sector view.

In the hygiene sector (Figure 5), the apprentice has access to liquid soap and alcohol gel dispensers, the sink, the paper towel dispenser, and the discard box. Next, in the vaccine preparation sector (Figure 6), the apprentice must select the corresponding needle size (from a menu with several options), the vaccine dose to be administrated, and pick the respective vaccine flask from the thermal box.

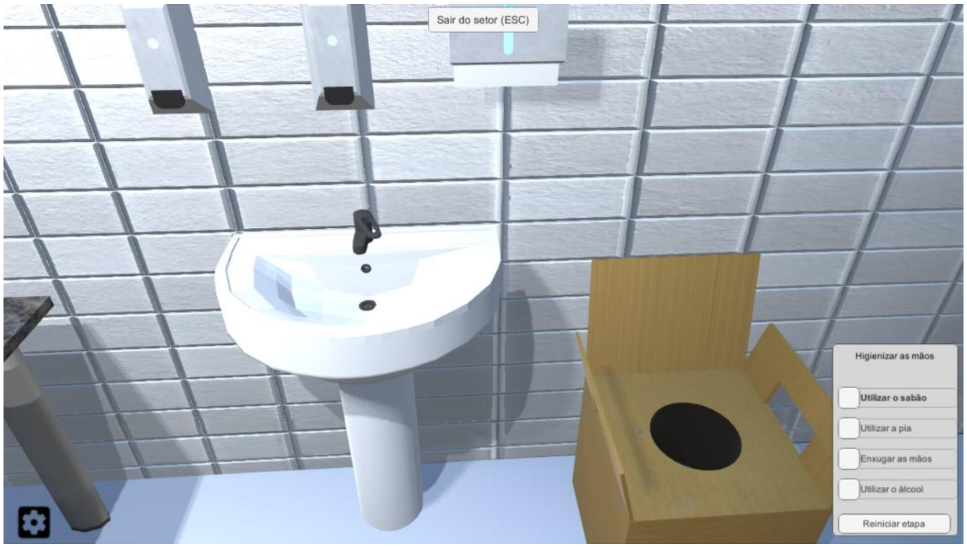

Figure 5: Hygiene sector view.

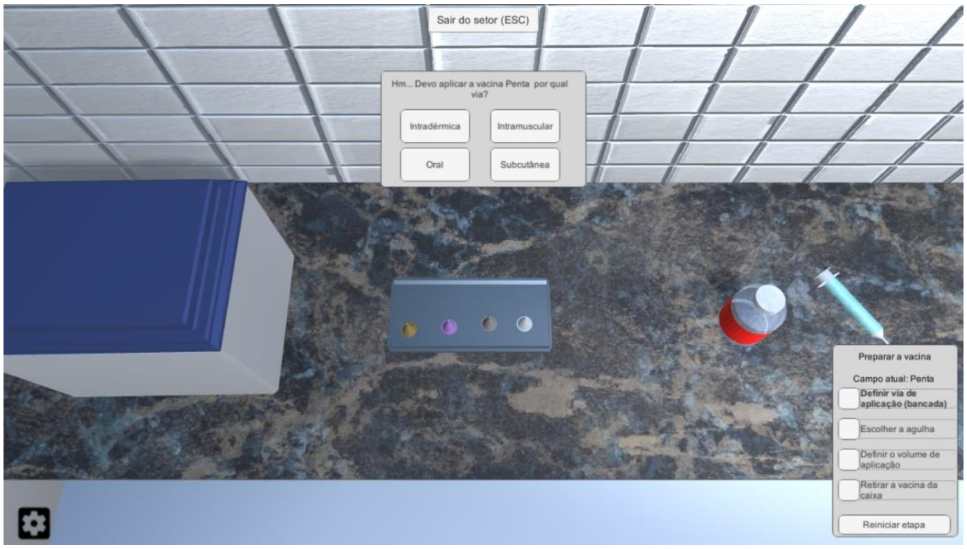

Figure 6: Vaccine preparation sector view.

The thermal box contains most vaccines presented in the Brazilian 2020 schedule. Thus, in a standard scenario, the apprentice must select a vaccine from 18 options, as listed in Table 1. The vaccines not presented in this list are not available through typical scenarios. However, an instructor can set up new vaccines in custom scenarios, allowing simulation considering vaccines with unique characteristics.

Table 1: Vaccines available in the simulation and their respective needles size, typical dose, and administration route selected for each one.

Vaccine	Needles	Typical Dose	Administration route
BCG vaccine	13:4.5	0.1ml	Intradermic
Hepatitis B	20:5.5 or 25:0.7	0.5ml or 1.0ml	Intramuscular

IPV (Inactivated poliovirus)	20:5.5	0.5ml	Intramuscular
Rotavirus vaccine	Does not apply	1.5ml	Oral
DTP/Hib/Hepatitis B	20:5.5	0.5ml	Intramuscular
Pneumococcal 10v	20:5.5	0.5ml	Intramuscular
Meningococcal C	20:5.5 or 25:0.7	0.5ml	Intramuscular
Yellow fever vaccine	13:4.5	0.5ml	Subcutaneous
Tetra viral vaccine	13:4.5	0.5ml	Subcutaneous
OPV (Poliovirus vaccine, Oral)	Does not apply	2 drops	Oral
DTP (Diphtheria-Tetanus-Pertussis Vaccine)	20:5.5	0.5ml	Intramuscular
Td (Tetanus and diphtheria vaccine)	25:0.7	0.5ml or 1.0ml	Intramuscular
MMR (Measles, mumps and rubella vaccine)	13:4.5	0.5ml	Subcutaneous
HPV (Human papillomavirus vaccine)	20:5.5 or 25:0.7	0.5ml	Intramuscular
Varicella	13:4.5	0.5ml	Subcutaneous
Tdap	25:0.7	0.5ml	Intramuscular
Hepatitis A	20:5.5	0.5ml	Intramuscular
Meningococcal ACWY	20:5.5 or 25:0.7	0.5ml	Intramuscular

The software actively records user actions through the simulation process, in which the user can export the respective report as an external file. Considering that an incorrect action prevents the simulation flow, recorded as a wrong choice, it is worth mentioning that all vaccine combinations (Table 1) are accepted as valid input. Since the correct administration varies according to each vaccine requirement, considering the patient's age, muscle state, and even unique medical conditions, some scenarios might demand a detailed assessment of the report data.

The educational assessment is related to how well the apprentice executed the vaccination process, considering the number of incorrect interactions. Thus, concluding the simulation, the performance report displays the following items:

1. The last stage performed.
2. List of vaccines administrated.
3. List of vaccines defined as a future date in the patient vaccines card.
4. The number of incorrect selections or inputs when:
    a) Defining a vaccine to be administrated.

b) Defining a return date.
c) Interacting with the information system.
d) Interacting in the hygiene sector.
e) Defining the administration route (total and specific by vaccine).
f) Defining the needle size (total and specific by vaccine).
g) Defining the dose (total and specific by vaccine).
h) Selecting a vaccine flask from the thermal box (total and specific by vaccine).

Auxiliary module for instructors – Scenario management

The auxiliary system allows the instructor to specify and manage parameters to create (or edit) a simulation scenario for his students. This system also aids in examining the performance report. The scenario variable options, organized into three categories, are shown in Table 2.

Table 2: Scenario variable options for simulation.

**Basic parameters**	Patient's name
	Patient's birth date
	Patient's 3D model
	Companion's 3D model
	Scenario description
	Consultation date
	Opening dialog text
**Patient's health conditions**	Pre-existing diseases and allergies
	Medication being used
	Reactions to previous vaccine administrations
**Vaccination history**	Type of the vaccination card
	Expected return date
	Vaccines administrated previously
	List of possible vaccines to be administrated by the apprentice
	Permission to use special fields in the vaccination card (the apprentice can set any vaccine in this field)
	Permission to apply a vaccine that is not listed in the current schedule

The system provides seven avatars (i.e., 3D-character models) and five different vaccination cards to grant more realism and educational possibilities. Besides the current vaccination card, the system provides four other card models previously used in Brazil. Figure 7 and Figure 8 represent the current vaccination card: the real-life card and the implemented version in the simulation, respectively.

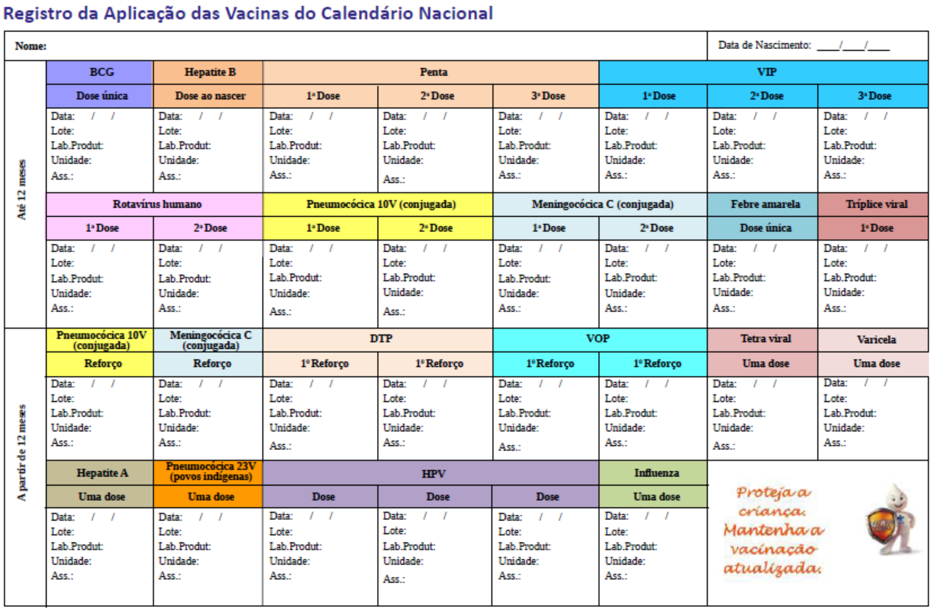

Figure 7: Current vaccination card (first page) used nowadays in Brazil.

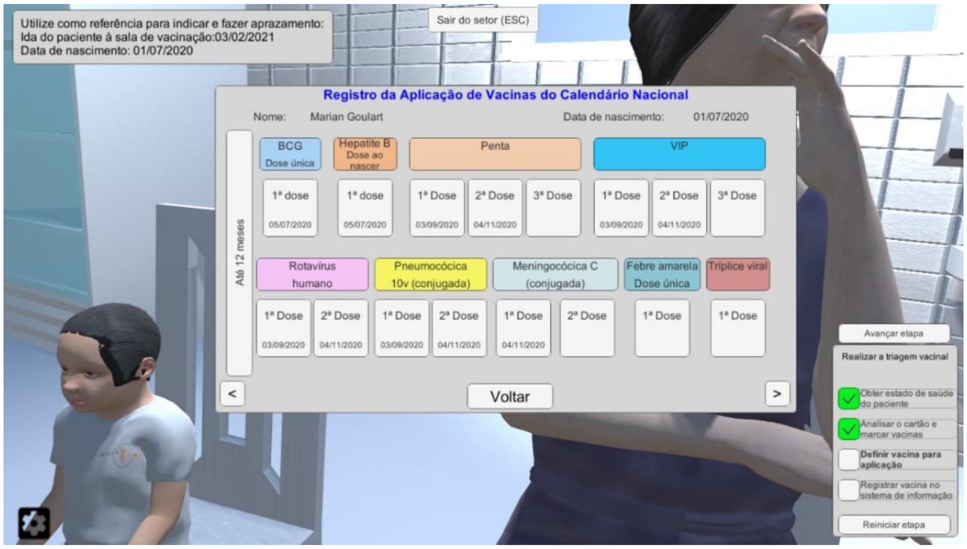

Figure 8: Implementation of the current vaccination card in the simulation (first page).
