# Supplementary material for: Software-Based Simulation on a 3D Environment for Vaccination Teaching and Learning: Design Science Research
Source: JMIR Med Educ. 2022 Dec 2;8(4):e35712. doi: 10.2196/35712 (PMC9758638; doi:10.2196/35712)
Supplement: Multimedia Appendix 2 [file mededu_v8i4e35712_app2.pdf]

Multimedia Appendix 2

Details of the use and technology acceptance evaluation

Table 1: Assessment of the measurement model.

Construct/Item	α > 0.7	ρa > 0.707	AVE > 0.5	VIF < 10	Loading > 0.7	Weight
**PE**	0.871	0.879	0.720			
PE1				2.412	0.866	0.317
PE2				1.915	0.816	0.348
PE3				3.809	0.795	0.265
PE4				5.767	0.912	0.253
**EE**	0.949	0.956	0.866			
EE1				4.346	0.913	0.227
EE2				3.995	0.921	0.291
EE3				5.528	0.949	0.284
EE4				5.374	0.940	0.271
**FC**	0.886	0.900	0.750			
FC1				2.321	0.771	0.261
FC2				3.305	0.878	0.282
FC3				2.820	0.833	0.274
FC4				7.676	0.969	0.333
**HM**	0.951	0.951	0.911			
HM1				3.608	0.933	0.362
HM2				9.064	0.970	0.346
HM3				7.834	0.960	0.340
**PV**	0.862	0.948	0.777			
PV1				2.025	0.810	0.267
PV2				2.908	0.912	0.355
PV3				2.230	0.919	0.501
**BI**	0.937	0.952	0.888			
BI1				2.974	0.900	0.301
BI2				7.370	0.969	0.379
BI3				6.123	0.956	0.378

Table 2: Discriminant assessment according with the Fornell-Larcker criterion.

	FC	PE	EE	HM	PV	HI	Sex	Age	BI
FC	**0.866**								
PE	0.604	**0.849**							
EE	*0.927*	0.776	**0.931**						
HM	0.753	0.798	0.800	**0.954**					

PV	0.812	0.516	0.682	0.769	**0.882**				
HI	-0.243	-0.411	-0.187	-0.232	-0.004	**1.000**			
Sex	0.256	0.233	0.230	0.321	0.262	-0.351	**1.000**		
Age	-0.366	-0.322	-0.311	-0.436	-0.449	-0.177	0.232	**1.000**	
BI	0.667	0.801	0.757	0.699	0.634	-0.244	0.265	-0.522	**0.942**

Table 3: Total indirect effects.

**Relation**	**Specific indirect effect**	***P* value**
HI → BI	0.102	.568
Sex → BI	0.233	.671
Age → BI	-0.402	.234

Table 4: R squared value calculated to the model's constructs.

**Construct**	**R squared**
FC	0.001
PE	0.103
EE	0.194
HM	0.119
PV	0.078
BI	0.738
